# High Basal Activity of the PTPN22 Gain-of-Function Variant Blunts Leukocyte Responsiveness Negatively Affecting IL-10 Production in ANCA Vasculitis

**DOI:** 10.1371/journal.pone.0042783

**Published:** 2012-08-03

**Authors:** Yali Cao, Jiajin Yang, Kerry Colby, Susan L. Hogan, Yichun Hu, Caroline E. Jennette, Elisabeth A. Berg, Youkang Zhang, J. Charles Jennette, Ronald J. Falk, Gloria A. Preston

**Affiliations:** 1 Division of Nephrology and Hypertension, UNC Kidney Center, Department of Medicine, University of North Carolina at Chapel Hill, Chapel Hill, North Carolina, United States of America; 2 Department of Pathology and Laboratory Medicine, University of North Carolina at Chapel Hill, Chapel Hill, North Carolina, United States of America; 3 Department of Medicine, Institute of Nephrology, Peking University First Hospital, Peking University, Beijing, People's Republic of China; University of Michigan Medical School, United States of America

## Abstract

Consequences of expression of the protein tyrosine phosphatase nonreceptor 22 (PTPN22) gain-of-function variant were evaluated in leukocytes from patients with anti-neutrophil cytoplasmic autoantibody (ANCA) disease. The frequency of the gain-of-function allele within the Caucasian patient cohort was 22% (OR 1.45), compared to general American Caucasian population (16.5%, *p* = 0.03). Examination of the basal phosphatase activity of PTPN22 gain-of-function protein indicated persistently elevated activity in un-stimulated peripheral leukocytes, while basal activity was undetectable in leukocytes from patients without the gain-of-function variant. To examine consequences of persistently high PTPN22 activity, the activation status of ERK and p38 MAPK were analyzed. While moderate levels of activated ERK were observed in controls, it was undetectable in leukocytes expressing PTPN22 gain-of-function protein and instead p38MAPK was up-regulated. *IL-10* transcription, reliant on the ERK pathway, was negatively affected. Over the course of disease, patients expressing variant PTPN22 did not show a spike in *IL-10* transcription as they entered remission in contrast to controls, implying that environmentally triggered signals were blunted. Sustained activity of PTPN22, due to the gain-of-function mutation, acts as a dominant negative regulator of ERK activity leading to blunted cellular responsiveness to environmental stimuli and expression of protective cytokines.

## Introduction

Anti-neutrophil Cytoplasmic Autoantibody (ANCA) disease is multifactorial in origin, as with many autoimmune diseases, involving complex interactions of genetic polymorphisms, epigenetic changes and environmental influences [1]–[5]. The list of genes associated with ANCA disease includes one generalized to autoimmune propensity, the protein tyrosine phosphatase non-receptor 22 (*PTPN22*) [6]–[24]. In 2004, a single nucleotide polymorphism (SNP) in the *PTPN22* gene was identified that resulted in a protein modification, which disrupted the regulatory domain of the phosphatase conferring a gain-of-function phenotype [25], [26]. The following year this genetic variant was linked with proteinase 3(PR3)-ANCA disease in a cohort of patients from Germany [24] and then in 2009 a similar association was made in a study of a cohort from Great Britain [27]. Now considered an autoimmunity-predisposing allele, this polymorphism strongly correlates with numerous other autoimmune diseases including type 1 diabetes (T1D) [28]–[32], rheumatoid arthritis (RA) [25], [33]–[38], systemic lupus erythematosus (SLE) [26], [39]–[41], Graves' disease [42], [43], and generalized vitiligo [44].

The aim of the studies presented here is to investigate effects of the gain-of-function variant on signaling responses in leukocytes from patients with ANCA disease and how these influence immunological events. ANCA have two primary targets, PR3 and myeloperoxidase (MPO), which are expressed solely on the surface of neutrophils and monocytes. Because PTPN22 is uniquely expressed in hematopoietic cell types, studies of the gain-of-function polymorphism are fundamentally important in this disease [25]. Binding of ANCA to its antigens stimulates cellular signal transduction pathways causing changes in gene transcription, cell activation status, and ultimately, neutrophil degranulation [45]–[49]. It is the aberrant release of neutrophils' noxious constituents that causes inflammation of vessel walls and injury of highly vascularized organs such as the kidney and lung [50]–[53].

What makes a gain-of-function variant of *PTPN22* particularly interesting, especially in the framework of multifactorial systemic autoimmune diseases like ANCA disease, is that protein tyrosine phosphatases (PTPs) serve as “sensors” and “transmitters” for environmental signals [54]. Alterations in genes encoding protein tyrosine phosphatases broadly affect kinase-phosphatase systems with deleterious effects on cellular equilibrium. PTPN22 is known to modulate the activity of the RAS and SRC-family signaling pathways, both of which are major pathways involved in immune modulation [55], [56]. Due to the proximal position of the RAS and SRC-family in numerous signal transduction cascades, including extracellular signal-regulated kinase (ERK), JNK, and p38 MAPK [57], inappropriate regulation would impact immune cell functions, including those emanating from integrins, Fc receptors, growth factor receptors, and cytokine receptors [58]–[61].

Intuitively, a function-altering, genetic polymorphism in *PTPN22* coupled with environmental exposures would place an individual at a higher risk for developing autoimmune disease. Environmental factors known to impact ANCA disease, at both disease onset and relapse, include bacterial and viral infections [62]–[64], aging [2], [65], seasonal changes [66] and silica exposure [3]. We have evidence that one manifestation of these factors is perturbation of epigenetic regulation of gene transcription. We found that gene silencing marks were altered in leukocytes of patients with ANCA disease resulting in aberrant transcription at the gene locus for PR3 and MPO [4], [67]. The gain-of-function variant could also be deviant in transmission of environmentally-induced epigenetic signals. For example, the JmjC-domain containing histone demethylase, JMJD3, is “induced” when the cell “senses” bacterial products and inflammatory cytokines within the microenvironment [68], [69]. We draw particular attention to JMJD3 because mRNA levels were abnormally high in ANCA disease patients concurrent with loss of epigenetic methylation marks at *PRTN3* and *MPO* loci [4].

Here we describe a study demonstrating how a genetic polymorphism can disrupt “sensors” of the signaling milieu. The data indicate that the PTPN22 gain-of-function variant confers abnormally high basal phosphatase activity perturbing proper responses to external stimuli in circulating neutrophils and lymphocytes of patients with ANCA disease.

## Materials and Methods

### Patients and clinical analysis

Patients with biopsy-proven ANCA disease enrolled in this study were diagnosed between 1985 and 2009, and followed in a life-long registry by physicians in the Glomerular Disease Collaborative Network (GDCN). Methods of identifying and enrolling patients in the GDCN have been described [65], [70], [71]. All study materials were given Institutional Review Board approval for human subjects' research (IRB study #97-0523) by the UNC-CH Office of Human Research Ethics. Study subjects gave informed, written consent and participated according to UNC Institutional Review Board guidelines. A total of 230 Caucasian patients with ANCA disease participated in the *PTPN22* genotyping study. Patients were categorized by diagnosis: granulomatosis with polyangiitis (GPA) [72]–[74], microscopic polyangiitis (MPA), Churg-Strauss syndrome (CSS), and renal-limited disease (Lim) [75], [76]. ANCA serotypes were determined by indirect immunofluorescence and/or antigen-specific PR3 and MPO enzyme-linked immune-absorbent assays (ELISA) (Invitrogen, Carlsbad, CA, USA) [77], [78]. Of 230 Caucasian ANCA-patients, 107 were PR3-ANCA and 109 were MPO-ANCA patients; 74 patients were diagnosed with GPA, 110 with MPA, 40 with renal limited, three with CSS, two with pulmonary capillaritis and one with neuro-limited disease. The Birmingham Vasculitis Activity Score (BVAS) 2003 version was used to rank disease severity activity: remission (BVAS = 0), active+ (BVAS 1–4), active++ (BVAS 5–9) and active+++ (BVAS≥10).

### PTPN22 genotyping

Genomic DNA was extracted from leukocytes in EDTA-treated blood using the Puregene DNA Purification System (Puregene, Minneapolis, MN, USA). DNA quality was spectrophotometrically determined by OD 260/280 nm ratios and by agarose gel visualization. Genotyping for SNP *C1858T* (rs2476601) and *G788A* (rs33996649) was performed using TaqMan-SNP-Genotyping Assay (Applied Biosystems, Foster City, CA). The primer sequences for *G788A* were: forward 5′ TTTGAACTAATGAAGGCCTCTGTGT 3′ and reverse 5′ ATTCCTGAGAACTTCAGTGTTTTCAGT 3′. The specific minor groove binder probe sequences were 5′ TTGATCCGGGAAATG 3′ (FAM) and 5′ TTGATCCAGGAAATG 3′ (VIC). The primer and the specific minor groove binder probe sequences for *C1858T* were commercially available and pre-designed by Applied Biosystems. TaqMan was performed by ABI PRISM 7900HT sequence detection system (Applied Biosystems).

### PTPN22 (lymphoid tyrosine phosphatase) activity assay

For phosphatase activity, total leukocytes were obtained after lysis of erythrocytes using RBC Lysis Buffer (NH_4_Cl) [79]. Patients analyzed included those with the gain-of-function variant (n = 12), non-variant (n = 12) and loss-of-function (n = 3). For analysis of specific cell types, neutrophils and lymphocytes&monocytes were separated from blood by Plasmagel (ZeptoMetrix, Buffalo, NY, USA) and Histopaque 1077 (Sigma, St. Louis, MO, USA).

Microtiter plate wells were coated in duplicate with mouse anti-human PTPN22 antibody (Abnova, Taipei, Taiwan) at a 1∶100 dilution and incubated overnight at 4°C. Normal mouse IgG served as a mock control. Leukocytes were lysed in lysis buffer (20 mM Tris-HCl, 150 mM NaCl, and 1 mM EDTA, pH 7.4, with 1 mM of phenylmethanesulphonylfluoride, 10 mg/ml of aprotinin, 10 mg/ml of leupeptin, 10 mg/ml of soybean trypsin inhibitor) at a concentration of 5×10^6^ cells/ml. Lysate was added into each pre-coated well (100 µl) and incubated for 3 hrs at room temperature (RT). After washing with lysis buffer without protein inhibitor, 100 µl of phosphatase substrate (*p*-NPP, Bio-Rad, Hercules, CA, USA) (in 100 mM Bis-Tris, pH 6.0, 5 mM DTT buffer) was added to each well [80]. Phosphatase activity was detected by a VersaMax Microplate Reader (Molecular Devices, Sunnyvale, CA, USA) at 405 nm.

PTPN22 protein was quantitated by capture enzyme-linked immunosorbant assay. Wells were coated with mouse anti-human PTPN22 antibody, or normal mouse IgG as a mock control. An aliquot of fresh cell lysate was added and PTPN22 protein detected with rabbit anti-PTPN22 (1∶200, Lifespan, Providence, RI, USA) and secondary antibody AKP-conjugated goat anti rabbit IgG (H+L) (1∶5000, Pierce, Rockford, IL, USA). For PTPN22-responsiveness studies, total leukocytes were pre-treated with PMA (100 ng/µl) as described (Sigma) for 10 mins at 37°C.

### Western blot method for ERK/pERK and p38/pp38 detection

Samples were analyzed from non-variant (n = 3), loss-of-function (n = 2) and gain-of-function (n = 4) ANCA patients. Fresh cell pellets were lysed in SDS sample buffer. Denatured protein was run on 8% Tris-HCl gel and transferred to nitrocellulose membrane (Schleicher and Schuell, Keene, NH, USA). After blocking, the membranes were incubated overnight at 4°C with appropriate dilutions of unconjugated primary antibodies, including mouse anti-human PTPN22 (Abnova), polyclonal anti-ERK (Abcam, Cambridge, MA, USA), polyclonal anti-phosphor-P44/42 MAP kinase (Cell Signaling, Danvers, MA, USA), polyclonal anti-P38 and polyclonal phosphor-P38 antibodies (Abcam). After washing, the membranes were incubated with horseradish peroxidase-conjugated goat anti-mouse or -rabbit IgG (H+L) (Jackson ImmunoResearch, West Grove, PA) for 1 h. Proteins were detected with Super-Signal West Pico Chemiluminescent Substrate (Pierce).

Densitometric scanning analysis of the ratio of intensity pERK/ERK and pp38/p38 was performed by ImageMaster VDS software.

### Analysis of microarray data

RNA was isolated from circulating leukocytes of gain-of-function (n = 4) and non-variant (n = 12) patients with ANCA disease [67], [79], [81]. The Affymetrix microarray gene chip was used for identification of gene expression levels, as previously described [67], [79], [81]. The data were then imported into the Partek Genomics Suite 6.4 program (Partek, St Louis, CA, USA) for an ANOVA statistical analysis and differentially expressed genes (≥2.0-fold, p-value <0.05) within the gain-of-function group were compared to non-variant group. The molecular network analysis was performed using Ingenuity Pathways Analysis (IPA) (Ingenuity Systerms, Redwood City, CA, USA) and the expression profile of genes from gain-of-function and non-variant groups was visualized using a principal components analysis (PCA) mapped scatter plot in Partek program.

### Taqman PCR analyses for IL-10 gene expression

*IL-10* primers and probes were purchased from Applied Biosystems. Fluorescence emission was monitored using the ABI PRISM 7900 HT sequence detection system. Relative level of total leukocyte RNA was determined by standard 2^(−ΔΔCt)^ calculations and expressed as fold change of reference control samples. Cytochrome c oxidase subunit 5B (COX5B) was used as a RNA loading standard [67], [81], [82].

### Statistical analysis

Differences in genotyping tests between ANCA patients and controls were analyzed by chi-square test. The direction and strength of these differences were assessed by calculating odds ratios. All of the alleles detected in our study were tested for the Hardy-Weinberg equilibrium. Clinical comparisons between patients with and without *C1858T* SNP for categorical measures were performed using chi-square tests. Continuous measures were compared using Wilcoxon rank sum test. A corrected *p*-value of <0.05 was considered significant. Wilcoxon Two-Sample test were used for comparisons of continuous measures and paired data were analyzed by the Signed Rank Test. A corrected *p*-value of <0.05 was considered significant. All statistical analyses were performed using SAS statistical program (SAS Institute, Inc., Cary, NC, USA).

## Results

### Identification of patients with the risk-associated allele of PTPN22

The gain-of-function allelic variant has a SNP changing a cytosine to a thymine (*C1858T*) which converts the codon from one coding for arginine (R) to one for tryptophan (W) (R620W), and this amino acid change confers a gain-of-function phenotype. A total of 230 Caucasian patients were genotyped for the risk-associated allele of *PTPN22 (C1858T)* using a TaqMan-SNP-Genotyping Assay. Different from cohorts studied in previous reports, the patient cohort studied here included both PR3- and MPO-ANCA groups. There was a significant association of the *C1858T* SNP allele in patients of Caucasian descent (22.2%) compared to the general American Caucasian population frequency (16.5%, *p* = 0.03) [32], with an odds ratio (OR) of 1.45 (95% confidence interval 1.02–2.04) (Table 1). The frequency was significantly higher in patients with a PR3-ANCA serotype (24.3%, *p* = 0.03, OR 1.63, 95% confidence interval 1.02–2.60) compared to American Caucasian population, but not in those with a MPO-ANCA serotype (20.2%, *p* = 0.32) (Table 1). Carriage of the variant allele had no influence on a diagnosis of GPA, (23.6%, *p* = 0.11) or with MPA (21.6%, *p* = 0.16) compared to the general American Caucasian population.

**Table 1:** Frequency of PTPN22 risk-allele (*T1858*) genotype in Caucasian ANCA patients.

	C/C	C/T+T/T	OR (95%CI)	*p-value*
All Patients	179(77.8%)	51(22.2%)	1.45 (1.02–2.04)	**0.03**
*PR3-ANCA	81(75.7%)	26(24.3%)	1.63 (1.02–2.60)	**0.03**
*MPO-ANCA	87(79.8%)	22(20.2%)	1.28 (0.78–2.10)	0.32

# Zheng W, 2005 (35).

* Excluded from analysis: 6 ANCA-neg; 5 PR3+MPO dual serology: 3 p-ANCA+ANA positives.

For completeness, genotypic analysis was performed to determine the frequency of the loss-of-function variant in PTPN22 (G788A, rs33996649) in the patient cohort. This polymorphism results in an amino acid change in residue 263 from arginine (R) to glutamine (Q) (R263Q) conferring a loss-of-function phenotype, and has been proposed to have a protective effect in SLE [80]. The frequency of this allele in ANCA patients was similar to the general American Caucasian population (4.8% vs 3.0%, *p* = 0.21) (Table 2) [80].

**Table 2:** Frequency of PTPN22 of protective allele (*A788*) in Caucasian ANCA patients.

	G/G	G/A+A/A	OR (95%CI)	*p-value*
Reference Controls	550(97.0%)	17(3.0%)		
Patients	219(95.2%)	11(4.8%)	0.62 (0.28–1.33)	0.21

Combining the reported frequencies of the risk allele, *C1858T*, in ANCA disease [24], [27] with the frequency observed in this USA cohort, a meta-analysis was performed. Even with the population differences, the combined odds ratio was 1.49 (95% confidence interval 1.28–1.73) (p<0.0001) (Table 3).

**Table 3:** Meta-analysis of the frequency of the *PTPN22 C1858T* SNP in ANCA Disease.

		CT+TT (%)	CC (%)	OR (95% Cl)	*p-value*
British cohort [27]				1.45(1.20–1.76)	0.0001
	ANCA	155 (24.76%)	471(75.24%)		
	Controls	1368 (18.46%)	6044(81.54%)		
German cohort [24]				1.71(1.15–2.54)	0.0078
	ANCA	57 (28.64%)	142(71.36%)		
	Controls	76(19.05%)	323(80.95%)		
USA cohort				1.45(1.02–2.04)	0.0368
	ANCA	51 (22.17%)	179(77.83%)		
	Controls	194 (16.47%)	984(83.53%)		
Total				1.49(1.28–1.73)	<0.0001
	ANCA	263(24.93%)	792(75.07%)		
	Controls	1638(18.22%)	7351(81.78%)		

### Assessment of functional changes attributed to the gain-of-function variant of PTPN22

**Figure 1:**
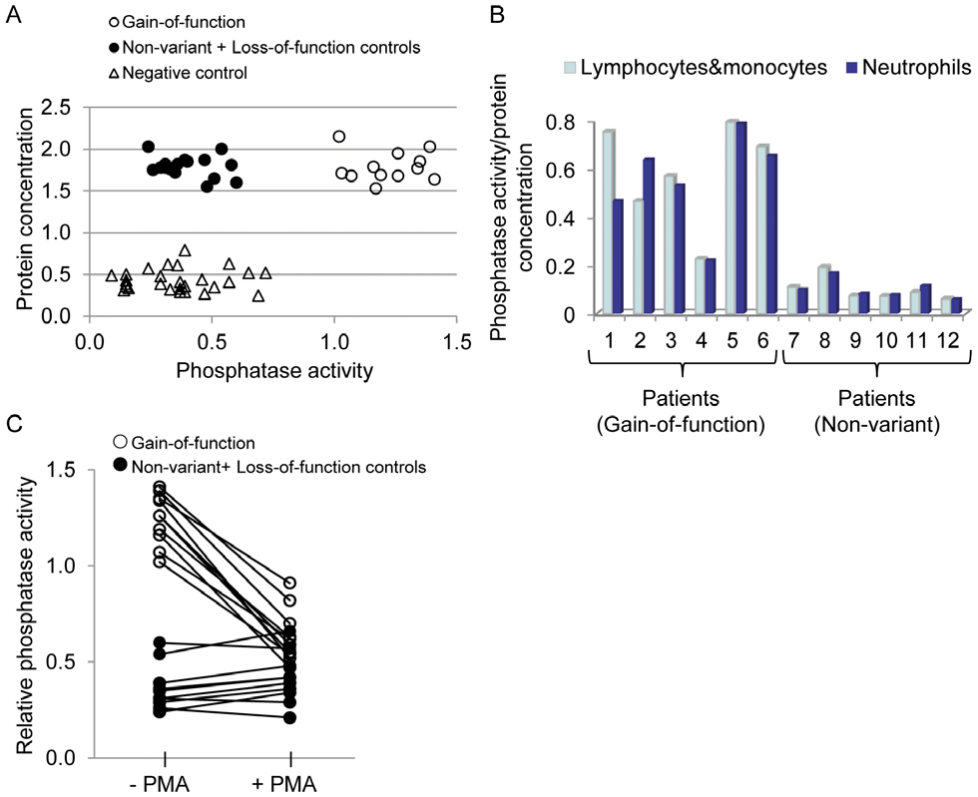
PTPN22 phosphatase activity in leukocytes. Basal level of PTPN22 phosphatase activity was high in leukocytes expressing the gain-of-function variant, (A) PTPN22 protein was active in all samples with the gain-of-function PTPN22 (R620W), while activity was undetectable in non-variant and loss-of-function control groups (*p*<0.0001). Activity values were plotted against total protein captured on ELISA plate using mouse-anti-PTPN22 antibody. For mock-controls, ELISA wells were coated with normal mouse IgG in parallel (B) High basal PTPN22 phosphatase activity was present in neutrophils (*p* = 0.0004) and lymphocytes&monocytes (*p* = 0.0003) (activity calculated as fold-increase above controls). (C) High basal phosphatase activity was significantly down-regulated after PMA treatment (*p*<0.0001), while there were no changes in non-variant controls (*p* = 0.75).

Based on the assertion that the PTPN22 variant (R620W) confers a gain-of-function phenotype, we hypothesized that unstimulated peripheral leukocytes from patients carrying this allele would have higher basal activity. Evaluations included both patients in remission and with active disease (Table 4). To determine amount of activity/protein concentration, PTPN22 protein was captured from total leukocyte lysates using an anti-PTPN22 antibody on two separate micro-titer plates. One was analyzed for total protein captured and the other for activity status of the captured protein. All patients with the gain-of-function variant (R620W) (n = 12) expressed high basal PTPN22 phosphatase activity in un-stimulated leukocytes, in stark contrast to controls with undetectable activity, including both leukocytes expressing the loss-of-function alleles (n = 3) and non-variant alleles (n = 12) (1.22±0.14 versus 0.41±0.12, *p*<0.0001) (Figure 1A). High basal phosphatase activity was present in R620W neutrophils (n = 6) (5.87±1.50 versus 1.04±0.38, *p* = 0.0004) as well as lymphocytes&monocytes (n = 6), but not in non-variant controls (n = 6) (*p* = 0.0003) (Figure 1B).

**Table 4:** Characteristics of patients with ANCA disease enrolled in the functional studies.

Patient/	Age	Gender	Diagnosis	ANCA	Disease
Genotype				subtype	activity
Gain-of-function					
P03*	70	F	GPA	PR3-ANCA	remission
P06*	35	M	GPA	PR3-ANCA	active+
P07*	63	F	MPA	PR3-ANCA	remission
P08*	25	F	MPA	PR3-ANCA	remission
P09	55	F	GPA	PR3-ANCA	active++
P12	33	F	MPA	MPO-ANCA	remission
P17	52	M	Lim	MPO-ANCA	active+
P19	58	M	GPA	PR3-ANCA	remission
P20	61	F	MPA	MPO-ANCA	active+
P21	57	F	Lim	MPO-ANCA	remission
P24	74	F	GPA	ANCA-Neg	active++
P27	55	M	GPA	PR3-ANCA	remission
Loss-of-function					
P02*	76	M	GPA	PR3-ANCA	remission
P05*	56	M	GPA	PR3-ANCA	remission
P25	61	M	GPA	PR3-ANCA	active++
Non-variant					
P01*	56	F	MPA	MPO-ANCA	remission
P04*	73	M	GPA	MPO-ANCA	remission
P10*	86	M	MPA	MPO-ANCA	remission
P11	21	M	GPA	PR3-ANCA	active+
P13	54	M	Lim	MPO+PR3	active+
P14	42	F	CSS	MPO-ANCA	active++
P15	45	F	GPA	PR3-ANCA	active+
P16	60	M	GPA	PR3-ANCA	active++
P18	34	M	GPA	PR3-ANCA	remission
P22	51	F	MPA	PR3-ANCA	active+
P23	59	F	GPA	PR3-ANCA	active+
P26	78	F	Lim	MPO-ANCA	active++

* Patient's sample included in western blot analysis of signaling pathways.

We asked if we could modulate this high basal phosphatase activity by treating the leukocytes with the powerful stimulant PMA (n = 10 with sufficient sample). PTPN22 phosphatase activity was significantly down-regulated (1.25±0.13 versus 0.64±0.14, *p*<0.0001) with the mean of decreases 0.61±0.13, while no change was observed in either the loss-of-function controls (n = 3) (0.27±0.04 versus 0.28±0.07, *p* = 0.07), with the mean of the decreases −0.01±0.08 or the non-variant controls (n = 7) (0.41±0.12 versus 0.47±0.11, *p* = 0.75) with the mean of the decreases −0.07±0.05 (Figure 1C). The data imply that constitutive phosphatase activity of the variant remains susceptible to pharmacological agents.

### Downstream effects of PTPN22 variant high basal activity

**Figure 2:**
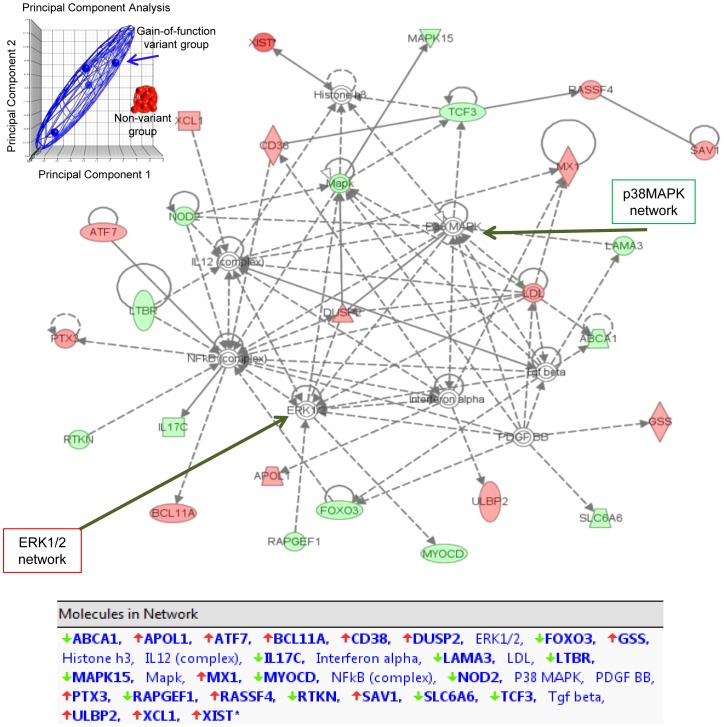
Bioinformatics analysis of Affymetrix microarray gene expression data, comparing leukocytes with the gain-of-function genotype to those with a non-variant genotype. Principal Component Analysis (PCA) scatter plot using Partek analysis is shown in the upper left corner. PCA is mathematically defined as an orthogonal linear transformation that transforms the data to a new coordinate system such that the greatest variance by any projection of the data comes to lie on the first coordinate (called the first principal component), the second greatest variance on the second coordinate, and so on. Each dot represents a patient's expression profile; the blue color dots represent gain-of-function and red show non-variant genotypes. Analysis using the Ingenuity Pathway Tools (IPA) software utilizes a repository of biological interactions and functional annotations created from millions of individually modeled relationships. The genes in red indicate increased expression and blue represents decreased expression, comparing gain-of-function with non-variant individuals. Primary networks identified were ERK1/2, p38MAPK, and NFκB networks.

To examine whether the high basal activity of the gain-of-function PTPN22 variant, observed in un-stimulated leukocytes, was affecting primary signaling pathways, we mined our existing Affymetrix array database. Four of the patients enrolled in that study were carriers of the gain-of-function *PTPN22* variant (Table 5). Comparisons between groups indicated that the high basal activity of the PTPN22 variant caused global changes in gene transcription. Analysis identified that 151 genes (98 up and 53 down) were differentially regulated (≥2.0-fold and p<0.05). Bioinformatic analysis using principal component analysis (PCA) showed remarkably different gene expression profiles comparing leukocytes with the gain-of-function genotype compared with non-variants (Figure 2). Genes with correlated expression profiles tend to cluster tightly into a small-size elliposoid by the wire mesh, while genes with less similar expression profiles form a looser cluster with a larger size of ellipsoid (Figure 2). The analysis indicates that there are dramatic intrinsic differences in signaling pathways associated with the gain-of-function polymorphism. Analysis using the Ingenuity Pathway Tools (IPA) software indicated that the primary networks affected were those involving ERK, p38MAPK and NFκB (Figure 2).

**Table 5:** Characteristics of patients with ANCA disease enrolled in the Affymetrix microarray study.

Patient/	Age	Gender	Diagnosis	ANCA	Disease
Genotype				subtype	activity
Gain-of-function					
1	26	F	MPA	MPO-ANCA	remission
2	45	F	MPA	MPO-ANCA	active+
3	54	F	MPA	MPO-ANCA	active++
4	54	F	MPA	MPO-ANCA	active++
Loss-of-function					
5	68	M	GPA	PR3-ANCA	active++
Non-variant					
6	71	M	GPA	PR3-ANCA	remission
7	61	F	MPA	MPO-ANCA	active+
8	56	M	GPA	PR3-ANCA	active++
9	55	M	GPA	PR3-ANCA	active++
10	38	M	MPA	PR3-ANCA	active++
11	60	F	MPA	MPO-ANCA	active++
12	55	M	GPA	PR3-ANCA	active++
13	72	M	Lim	PR3-ANCA	active+++
14	79	M	MPA	MPO-ANCA	active+++
15	60	M	MPA	PR3-ANCA	active+++
16	17	F	MPA	PR3-ANCA	active+++

**Figure 3:**
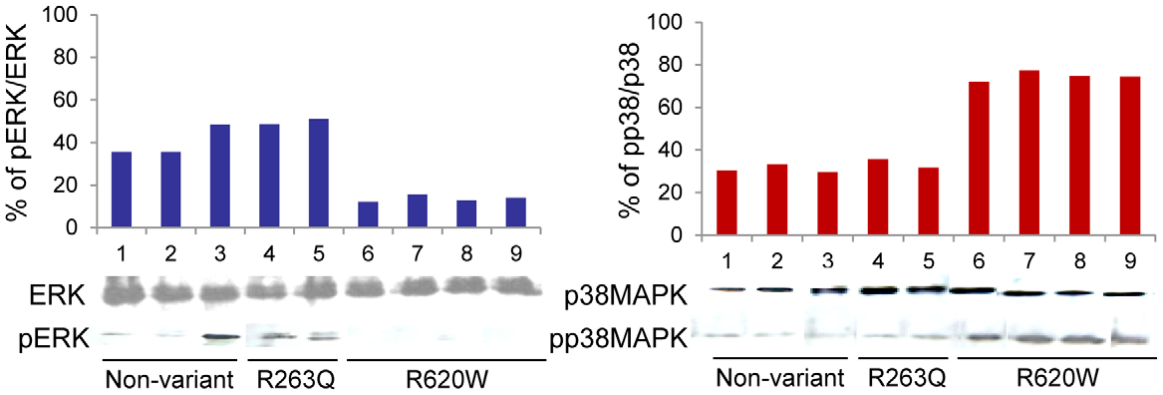
Analysis of ERK1,2 and p38MAPK phosphorylation status. Bar graphs represent the ratio of intensity pERK/ERK and pp38/p38MAPK as quantitated by densitometric scanning analysis using ImageMaster VDS software. Western blot analysis for ERK1,2 and p38MAPK activation demonstrates PTPN22 gain-of-function (R620) exerts a negative effect on the ERK signaling pathway, compared to loss-of-function (R263Q) and non-variant controls. In contrast p38 mitogen-activated protein kinase (p38 MAPK) was increased with the gain-of-function (R620) phenotype.

These data are consistent with reports that PTPN22 function regulates signaling molecules leading to activation of ERK1,2 [83], [84]. We examined the phosphorylation status of ERK1,2 in four patients with PTPN22 (R620W), two patients with PTPN22 loss-of-function allele (R263Q) and three patients with the normal allele (Table 4). Phosphorylated/active ERK was undetectable with PTPN22 gain-of-function activity, in contrast to controls. Instead, the phosphorylated/active p38 mitogen-activated protein kinase (p38 MAPK) form was elevated (Figure 3).

### IL-10 gene expression is down regulated in leukocytes with the PTPN22 gain-of-function (R620W) variant

**Figure 4:**
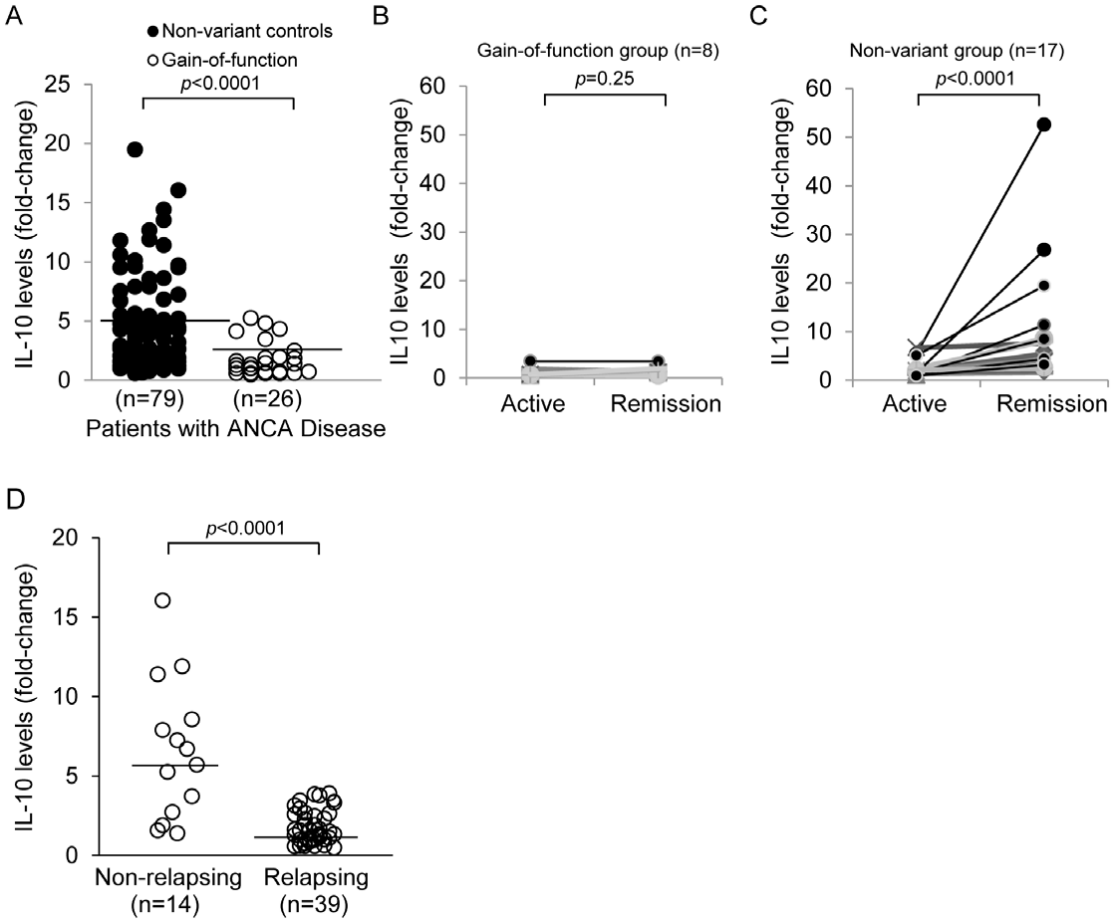
*IL-10* mRNA expression. (A) *IL-10* transcript levels are reduced in leukocytes from PTPN22 (R620W) positive patients. (A) IL-10 mRNA expression, which is mediated through the ERK pathway, was significantly lower in gain-of-function patients (*p*<0.0001) by quantitative TaqMan PCR. (B) Longitudinally, the baseline level of IL-10 message in patients with the gain-of-function variant did not increase as they transitioned from active disease to remission (*p* = 0.25). (C) In contrast, patients with normal PTPN22 showed a robust increase in IL-10 as they entered remission (*p*<0.0001). (D) Decreased IL-10 levels were associated with the relapsing group (n = 39, 1.8±1.15), and higher level in the non-relapse patient group (n = 14, 6.6±4.4, *p*<0.0001).

Maximal IL-10 production requires signaling through activated ERK and the downstream phosphorylation of the Sp1 transcription factor [85], [86]. Based on decreased activity of ERK with the gain-of-function (R620W) variant, we hypothesized the *IL-10* gene expression would be negatively affected. *IL-10* mRNA levels were significantly lower in patients with the gain-of-function variant (n = 26), as compared to non-variant controls (n = 79)(1.8±1.4 versus 5.0±4.1, *p*<0.0001) (Figure 4A). Longitudinally, baseline *IL-10* transcripts did not increase in patients having the gain-of-function genotype as their disease progressed from active disease (BVAS≥1) to remission (BVAS = 0) (n = 8) (1.17±1.01 versus 1.50±0.88, *p* = 0.25) with the mean of the increase 0.33±0.61 (Figure 4B). In contrast, patients without the SNP showed a robust increase in *IL-10* transcripts as remission was achieved (n = 17) (2.37±1.69 versus 10.19±12.78, *p*<0.0001) with the mean of the increase 7.82±12.01 (Figure 4C). Epidemiological analyses indicated that patients having gain-of-function (R620W) variant progressed to end-stage kidney disease (ESKD) on average 20 months faster (18% vs. 9%, *p* = 0.04). No substantial differences were found in regard to gender, ANCA serotype, disease diagnosis, treatment resistance, or organ involvement.

It was reported that lower IL-10 levels in remission are associated with a higher relapse rate in long-term follow-up [87]. Analysis of our cohort indicated that higher *IL-10* transcript levels were associated with non-relapsing disease (n = 14, 6.6±4.4, *p*<0.0001), while lower levels were associated with a relapsing-disease history (n = 39, 1.8±1.15) (Figure 4D). There were 14 patients in this study that had the gain-of-function SNP. Three (21%) were in the non-relapsing group while 11(79%) were in the relapsing-disease group.

## Discussion

This is the first report of studies on the basal activity of the PTPN22-gain-of-function protein in non-stimulated leukocytes, immediately following blood draw. High basal PTPN22 phosphatase activity was detected in leukocytes of every patient tested who had the *PTPN22* SNP *(C1858T)* genotype, while their non-variant counterparts had undetectable activity. We were intrigued that high basal PTPN22 phosphatase activity was present in neutrophils expressing the gain-of-function variant. Interestingly, one consequence was activation of the p38 MAPK pathway. P38 MAPK regulates macrophage and neutrophil functional responses, including respiratory burst activity, and chemotaxis. High activity was also present in the lymphocyte&monocyte pool which would support the findings of altered T cell function in Type-1 diabetes and Jurkat T leukemia cells overexpressing the transfected gain-of-function variant [80], [88]–[90].

**Figure 5:**
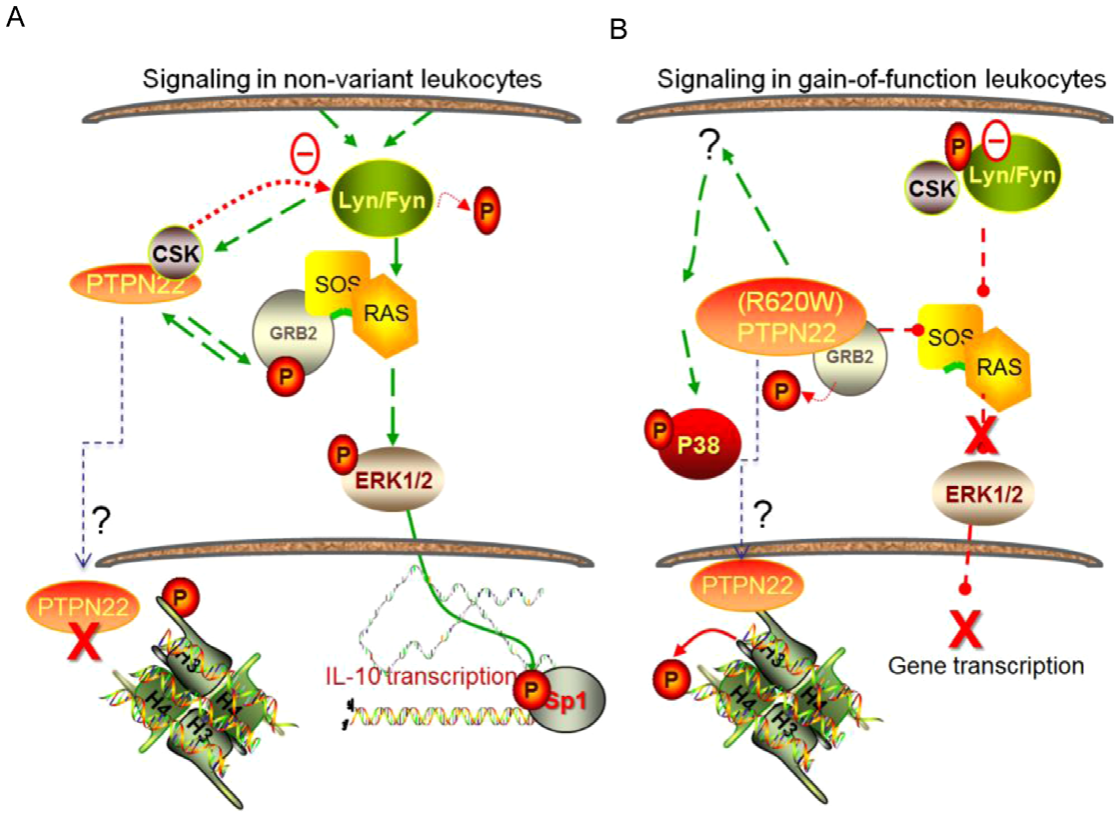
Signaling pathways disrupted by the gain-of-function variant of PTPN22. (A) Signaling pathways affected by PTPN22 include SRC-family kinases (Lyn/Fyn) and RAS pathways [83], [84]. It can affect the activity of SRC-family kinases through regulation of CSK (cSRC Kinase) [96], [97]. PTPN22 can affect RAS activity through binding to GRB-2 (Growth factor receptor-bound protein 2). ERK1,2 phosphorylates and activates many transcription factors, including the transcription factor Sp1 depicted here, which regulates the transcription of IL-10 [86]. (B) Changes in PTPN22 function due to the gain-of-function phenotype. PTPN22 (R620W) amino acid change lies within a domain that binds CSK, resulting in a greatly reduced binding [84]. Thus CSK is available for binding and inhibiting SRC. Also, a gain-of function alteration could act as a super-antagonist of epigenetic nucleosome remodeling, based on reports that PTPs directly dephosphorylate histone tails [98]. The gain-of-function phosphatase activity also affects the function of GRB2 restraint of RAS signaling and a loss of ERK1/2 phosphorylation/activation and loss of Sp1 transcriptional activity.

Constitutive activity of PTPN22 gain-of-function variant was associated with global changes in the transcriptome, inasmuch as the assessment of the small cohort studied here. Activity of normal PTPN22 is under regulatory constraints, one of which is inhibition by phosphorylation on Ser-35 by protein kinase C (PKC) [91]. The mechanistic pathway of PTPN22 is under study by many groups and to date, functional partners of normal PTPN22 include growth factor receptor-bound protein 2 (GRB-2), and C-SRC kinase (CSK) [89]. The schematic in Figure 5A illustrates some of the signaling pathways reportedly linked with PTPN22 function, and in Figure 5B a prediction – based on the data presented here – of how they may be perturbed by constitutively high PTPN22 activity.

It is reported that decreased IL-10 production during remission is a predictor of relapse in ANCA disease [87]. Results in our study are in agreement. Moreover, we found that *IL-10* message was lower in patients expressing the PTPN22 gain-of-function variant and that a high proportion of these fell into the relapsing-disease category. Reduced IL-10 responses in patients with the gain-of-function variant can be considered as a deviation in transmission of signals within the microenvironment of the body. We examined gene expression of the anti-inflammatory cytokine IL-10, because it is known to be responsive to environmental triggers [86], [92]; for example, neutrophils secrete high amounts of IL-10 in response to bacterial products [93]. Relevant to this discussion, expression of *IL-10* is regulated through epigenetic mechanisms at the *IL-10* locus through signals transmitted by the ERK signaling pathway, which is responsive to many cytokines, growth factors, and importantly environmental stress [85], [86], [94].

Although the allelic variant of *PTPN22* has been reported as a predisposing factor in ANCA disease [95], this is the first report of its frequency in a USA patient cohort. In this patient cohort, association of the disease-associated allele was skewed toward PR3-ANCA disease. In concordance, the geographic distribution of this variant allele is highest in countries where PR3-ANCA predominates with the highest in Finland (15.5%), Sweden (12%) and then UK (8%), decreasing southward to Spain (6%) and Italy (2%). Combined, these studies provide statistical power to support an association between the *PTPN22* variant and ANCA disease (OR 1.49, *p* = 4.15×10^−6^). The allele is nearly absent in African American and Asian populations [90].

PTPN22 integrates and transmits environmental changes through dynamic signalling molecules. Studies of the gain-of-function variant in multifactorial autoimmune diseases, such as ANCA disease, provides the opportunity to understand how disease outcome can be influenced by a complex interplay of genetic regulation and environmental influences.
